# Comparison of anti-inflammatory activity of serratiopeptidase and diclofenac in albino rats

**DOI:** 10.4103/0976-500X.72362

**Published:** 2010

**Authors:** Shilpa P. Jadav, Nilesh H. Patel, Tarang G. Shah, Maganlal V. Gajera, Hiren R. Trivedi, Bharat K. Shah

**Affiliations:** *Department of Pharmacology, M.P. Shah Medical College, Jamnagar - 361 008, Gujarat, India*; 1 *Drug Monitoring Research Institute, Rabale, Navi Mumbai - 400 701, India*; 2 *Accutest Research Laboratory (I) Ltd, Bodakdev, Ahmedabad - 380059, Gujarat, India*; 3 *Department of Pharmacology, College of Dental Education and Research Centre, Bopal, Ahmedabad, Gujarat, India*

Sir,

Acute and chronic inflammatory diseases are one of the most important health problems in the world. Although several agents are known to treat inflammatory disorders, their prolonged use often leads to gastric intolerance, bone marrow depression and water and salt retention.[1] Although the newer nonsteroidal anti-inflammatory drugs are more potent, having the same or greater action against many chronic clinical situations and also having a favorable safety profile as compared with the older groups, an entirely satisfactory solution is still eluding.[2] Serratiopeptidase, a proteolytic enzyme, has been found useful in patients suffering from acute or chronic inflammatory disorders of ear, nose or throat, such as laryngitis, catarrhal rhino-pharyngitis and sinusitis.[3] Hence, the present study was undertaken to compare anti-inflammatory effect of serratiopeptidase against diclofenac sodium.

Sixteen Charles foster albino rats of either sex weighing 150-250 g were randomly divided into four groups, with four animals in each group. The animals were maintained at room temperature and fed with standard pellet diet (Pranav Agro Industries Pvt. Ltd., Vadodara, India) and water, *ad libitum*. Diclofenac sodium (Novartis India Ltd., Mumbai, India), serratiopeptidase (Systopic Laboratories Ltd., New Delhi, India) and formaldehyde were used for the study. The experimental study protocol was approved by the Institutional Animal Ethics Committee (Registration number 454/1a/CPCSEA).

**Figure 1:**
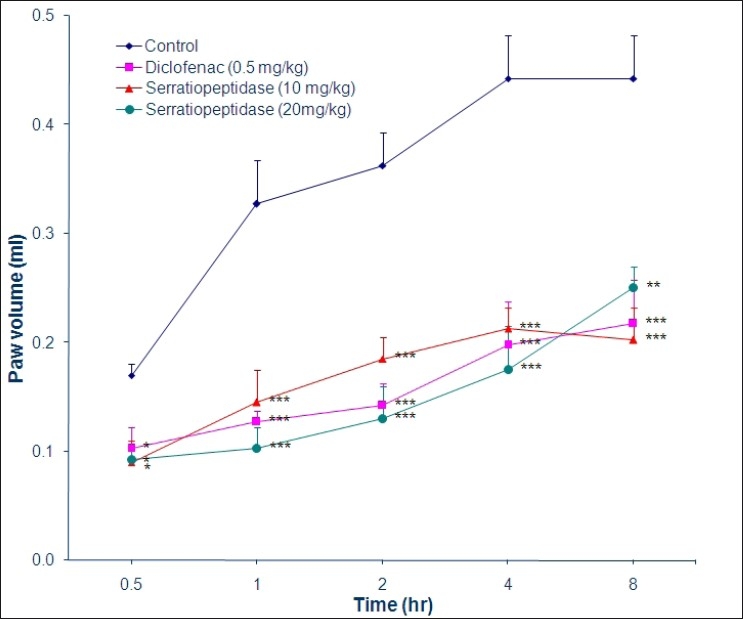
Anti-inflammatory effect of serratiopeptidase on acute inflammation induced by formalin in albino rats. Data are expressed as mean ± SEM (n=8 values in each group); One-way ANOVA followed by Tukey’s test; *P < 0.05, **P < 0.01, ***P < 0.001 compared with control

**Figure 2:**
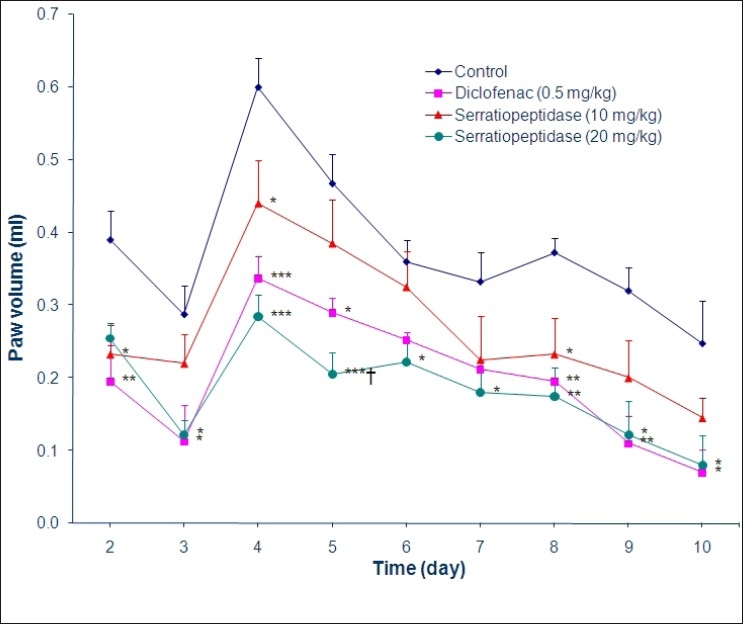
Anti-inflammatory effect of serratiopeptidase on chronic inflammation induced by formalin in albino rats. Data are expressed as mean ± SEM (n=8 values in each group); One-way ANOVA followed by Tukey’s test; *P < 0.05, **P < 0.01, ***P < 0.001 compared with control; ^†^*P* < 0.05 compared with serratiopeptidase (10 mg/kg)

Acute inflammation was induced by subplantar injection of 0.1 mL of 2% formalin in both hind paws, 1 h after oral administration of distilled water (0.4 mL/100 g) in group 1, which served as control, diclofenac (0.5 mg/kg) in group 2, which served as standard, serratiopeptidase (10 mg/kg) in group 3 and serratiopeptidase (20 mg/kg) in group 4. The paw volume was measured immediately and at 0.5, 1, 2, 4 and 8 h following the injection of formalin. For chronic inflammation study, the above animals were further treated with serratiopeptidase, diclofenac or distilled water twice daily orally for 9 consecutive days. A second injection of formalin was given on the third day.[4] The daily changes in the paw volume were measured by plethysmograph and was expressed as an increase in the paw volume. The results of acute and chronic inflammation are represented graphically in Figures 1 and 2, respectively.

All values are presented as mean ± SEM. Differences between means were assessed by one-way analysis of variance, followed by Tukey’s multiple comparison test using GraphPad Prism Version 5.01 software. *P* < 0.05 was considered statistically significant.

Serratiopeptidase (10 and 20 mg/kg) significantly inhibited acute inflammation of paw at 1, 2, 4 and 8 h after formalin injection (*P* < 0.0001), which was comparable with diclofenac sodium-treated group. Percent inhibition with serratiopeptidase (10 mg/kg), serratiopeptidase (20 mg/kg) and diclofenac (0.5 mg/kg) were 54.55%, 43.18% and 50% respectively 8 h after administration of formalin.

Serratiopeptidase showed significant inhibition of chronic inflammation of paw up to tenth day. On the tenth day serratiopeptidase (10 and 20 mg/kg) and diclofenac (0.5 mg/kg) significantly inhibited inflammation induced by formalin by 40%, 68% and 72% respectively (*P* = 0.018). This indicates that higher dose of serratiopeptidase (20 mg/kg) was more effective than low dose of serratiopeptidase (10 mg/kg) in reducing paw edema during chronic phase of inflammation.

Acute inflammation induced by formalin results from cell damage, which provokes the production of endogenous mediators such as histamine, serotonin, prostaglandins and bradykinin.[5] Serratiopeptidase hydrolyses bradykinin, histamine and serotonin responsible for edematic status.[6]

Despite some possible limitations of our study such as single model study, relatively small sample size and possible subjective error in the assessment of paw edema, the results of the present study indicate that serratiopeptidase is orally effective and possesses anti-inflammatory activity, which is nearly equivalent to diclofenac sodium in both acute and chronic phases of inflammation.
